# Implementing the Primary Care Behavioral Health model into routine practice in Swedish primary care: a pilot trial

**DOI:** 10.1186/s12913-026-15452-8

**Published:** 2026-09-03

**Authors:** Lise Bergman Nordgren, Kristin Thomas, Erica Skagius Ruiz, Kocher Koshnaw, Mats Fredriksson, Per Nilsen, Hanna Israelsson Larsen

**Affiliations:** 1 Department of Medicine, Faculty of Medicine and Health, Örebro University, Örebro, Sweden; 2 Division of Psychiatry, Region Örebro, Örebro, Sweden; 3 Department of Health, Medicine and Caring Sciences, Linköping University, Linköping, Sweden; 4 Primary Health Care Center, Region Östergötland, Linköping, Sweden; 5 Department of Experimental and Clinical Medicine, Linköping University, Linköping, Sweden; 6 School of Health and Welfare, Halmstad University, Halmstad, Sweden; 7 Wallenberg Centre for Molecular Medicine, Linköping University, Linköping, Sweden

**Keywords:** Primary care, Mental health, Implementation, Primary Care Behavioral Health, Clinical trial

## Abstract

**Background:**

Mental health problems are an increasing public health concern, and demand for care is exceeding available resources. The Primary Care Behavioral Health (PCBH) service delivery model shows promise for managing mental health problems in primary care, but its effects require further evaluation. This study examined how the implementation of PCBH in standard Swedish primary care was associated with accessibility of behavioral and mental health care and psychotropic prescription patterns.

**Methods:**

This pragmatic multicenter stepped-wedge clinical trial reports results from the first four pilot centers implementing PCBH in southeast Sweden. These centers had 37,100 listed patients. Study participants included all individuals receiving mental health care during the study period (*n* = 6413, mean age 50 years, 60% women). Data from electronic medical records were analyzed regarding behavioral health consultant (BHC) accessibility (number of appointments and individual patients) and prescriptions of psychotropic drugs (ATC codes: N05A, N05B, N05C, and N06A). Three time periods were compared: baseline (period 1), implementation (period 2), and the year after implementation (period 3).

**Results:**

After the implementation of PCBH, mean waiting times for a first BHC appointment decreased between 30% and 39%: from 16 days in period 1 to 10 (period 2) respectively 11 days (period 3), *p* < 0.001 both comparisons. Waiting times for all BHC appointments decreased between 21% and 24%; from 18 days (period 1) to 13.7 (period 2) respectively 14.2 days (after implementation), *p* < 0.001 both comparisons. The total number of appointments with a BHC increased between 21% and 34%; from 1762 (period 1) to 2359 (period 2) and then 2127 (period 3), *p* < 0.001 both comparisons. Individuals receiving care from a BHC increased between 16% and 42%; from 702 (period 1) to 995 (period 2) and 815 (period 3), *p* < 0.005 both comparisons. The total prescription of psychotropic drugs decreased between 14% and 18%; from 8025 (period 1) to 6570 (period 2) and 6927 (period 3) *p* < 0.001 both comparisons.

**Conclusions:**

These findings suggest that implementation of PCBH improves access to mental health care and reduces psychotropic prescriptions. However, the improved access to care and reduction in psychotropic medication declined somewhat the year after implementation. Future research should focus on longitudinal outcomes with the use of comparison groups and systematically include patient-reported outcomes, experience measures, and staff perspectives to assess effectiveness and implementation experiences.

**Trial registration:**

ClinicalTrials.gov (NCT05633940), registered 2021-04-21.

## Background

Mental health issues have posed significant global health challenges for decades, consistently ranking among the top ten contributors to the global burden of disease [1–4]. The World Health Organization reports that, globally, one in eight individuals has a mental health disorder; mood and anxiety disorders are the most prevalent [5]. Alarmingly, there has been no evidence in these reports of a decline in mental health challenges since 1990 [1]. The 2022 National Public Health Survey conducted by the Public Health Agency of Sweden found that 9% of respondents experienced severe psychological distress, reporting feelings of worry or hopelessness. For individuals, mental health problems can lead to reduced functioning and significantly contribute to absenteeism from work [6]. In Sweden, mental disorders are the leading cause of sickness absence, accounting for 50% of ongoing sick leave among women and 39% among men, as reported by the Swedish Social Insurance Agency [6].

Primary care is the first point of contact for most individuals with somatic and mental health problems [4, 5, 7, 8]; 50%–70% of those diagnosed with a mental disorder receive treatment in primary care [2, 9]. All primary care centers in Sweden are therefore obligated to have appropriate staffing even for the mental health needs. However, several challenges exist in this setting, including a shortage of qualified staff to properly address mental health issues, underutilization of psychological or behavioral interventions, long waiting times for care, and insufficient knowledge of various mental and biopsychosocial conditions among professionals and in the general population [2–4, 8, 10, 11]. The Organization for Economic Co-operation and Development (OECD) has emphasized the need to expand the competence of primary care professionals by improving mental health training, incorporating mental health specialists into primary care settings, and implementing clear referral and consultation practices with specialists [8]. Furthermore, the OECD underscores the importance of addressing workplace and sickness absence issues [8].

The Primary Care Behavioral Health (PCBH) service delivery model was developed in an effort to reduce barriers to seeking and receiving behavioral or mental health care [12, 13]. This model emphasizes the integration of mental health care into routine primary care settings, improving accessibility to psychological interventions through highly accessible, on-site behavioral health consultants (BHCs). The PCBH model aims to enhance the management of all patients, not only those directly seen by BHCs, and has been described as an interdisciplinary, team-based approach that addresses population health problems through a biopsychosocial framework [10]. Core components of the model include the use of brief, focused visits and a scheduling system that supports open access to BHCs to organize appointments directly or within a few days [10]. The primary objective is to identify and address behavioral and mental health issues at an early stage, preventing symptoms from escalating into severe conditions and seamlessly integrating mental health care into primary care, ensuring it is treated with the same importance as physical health. To achieve this, the model proposes providing patients in primary care with proactive, timely, and adequate treatment for both physical and mental health concerns [10, 13–15].

Integrating the PCBH model into regular primary care has been linked to reduced waiting times for treatment by a BHC and increased likelihood of patient engagement, resulting in a higher number of attended visits [16]. Some evidence of improved patient satisfaction and the acceptability of the model have also been reported [13, 17]. However, although the PCBH service delivery model is gaining popularity [14, 15, 18], there has been a call for larger trials and more robust scientific evidence [13, 15]. Research on the model’s impact on reduced reliance on secondary care, enhanced patient satisfaction and functioning, and the possible overuse of psychotropic medications for mental health problems remains limited. In addition, the impact of the PCBH model in different primary care settings needs to be investigated [15, 19]. No previous studies have reported results of implementing PCBH in Swedish primary care.

In Sweden, where BHCs already are either working within or are connected to all PCCs, the PCBH model has attracted the attention of clinicians and decision-makers, and it is currently being implemented in several regions responsible for delivering healthcare services in Sweden [20, 21]. This pilot study presents the results on management of mental health problems (i.e., waiting times for BHCs and physicians, and prescription of psychotropic drugs) in a population of adults 18 years or older from the first four primary care centers (PCCs) in Region Östergötland, Sweden, that integrated the PCBH service delivery model into routine practice. The results form part of a larger stepped-wedge trial. The setup for the broader study, including the project’s logic model, has been described in detail elsewhere [20].

### Aim

The aim of this pilot study was to investigate the effects of integrating the PCBH model in terms of accessibility to mental health care for adult patients with mental health problems and prescription patterns of psychotropic drugs. Data and insights served as support in planning a larger stepped-wedge trial.

## Materials and methods

### Study design and setting

In 2018, Region Östergötland, located in southeast Sweden, decided to offer support to facilitate the integration of the PCBH model into routine practice at all PCCs in the region. To investigate all aspects of this large-scale implementation, a pragmatic stepped-wedge cluster trial was designed [20]. In 2019, the first four pilot centers in the trial were set up to provide insights on scaling up implementation and to make sure the care model did not generate negative results on access to mental health care. This study does not cover implementation activities per se, nor staff knowledge or experiences on working according to the PCBH model. All data except for reports regarding full-time positions for medical staff were collected from electronic records and required no further action from the PCCs. This pilot study covers outcomes from these four centers.

The Swedish healthcare system is a decentralized model funded primarily by taxes. The state sets overall health policies and regulations and provides financial support to the regions. Sweden is divided into 21 regions, each self-governed and responsible for delivering healthcare services to its residents, including hospitals and primary care. All inhabitants in Sweden are listed to a primary health care center, and primary health care serves as the first point of contact for most symtoms and diseases for the whole population. In standard Swedish primary care, visits to a BHC typically last 45–60 min and are usually provided in a series of ten sessions, often after several weeks of waiting.

The study was approved by the ethics review authority (reference number 202005572) and registered in ClinicalTrials.gov (reference number NCT05633940).

### The Primary Care Behavioral Health model

The PCBH model is a model of service delivery stipulating that behavioral or mental health problems should be detected, addressed, and treated without delay. It is an interdisciplinary approach concerning all clinical staff but mental health providers (called behavioral health consultants [BHCs] within the PCBH model) play a significant part [22]. The BHCs are fully integrated within the health care team [22] and provide not only psychological interventions to the patients, but are also working closely with physicians and assisting all staff at the primary care centre. The BHCs belong to various professions (e.g., counselors or clinical psychologists) and the model includes six core components [10]. The BHC is described as: a *generalist* who treats all patients regardless of age and concern, working toward increased *access* to care. The model is *team-based* aiming for *high productivity* (i.e., by scheduling patients for focused 15–30 min appointments). The BHC also has a role in *education*, teaching both patients and staff about the need for a biopsychosocial approach. The model also stipulates that the BHC should be seen as a *routine* component in primary care, with a schedule that supports open access to BHCs on the same day.

### The implementation process

**Fig. 1:**
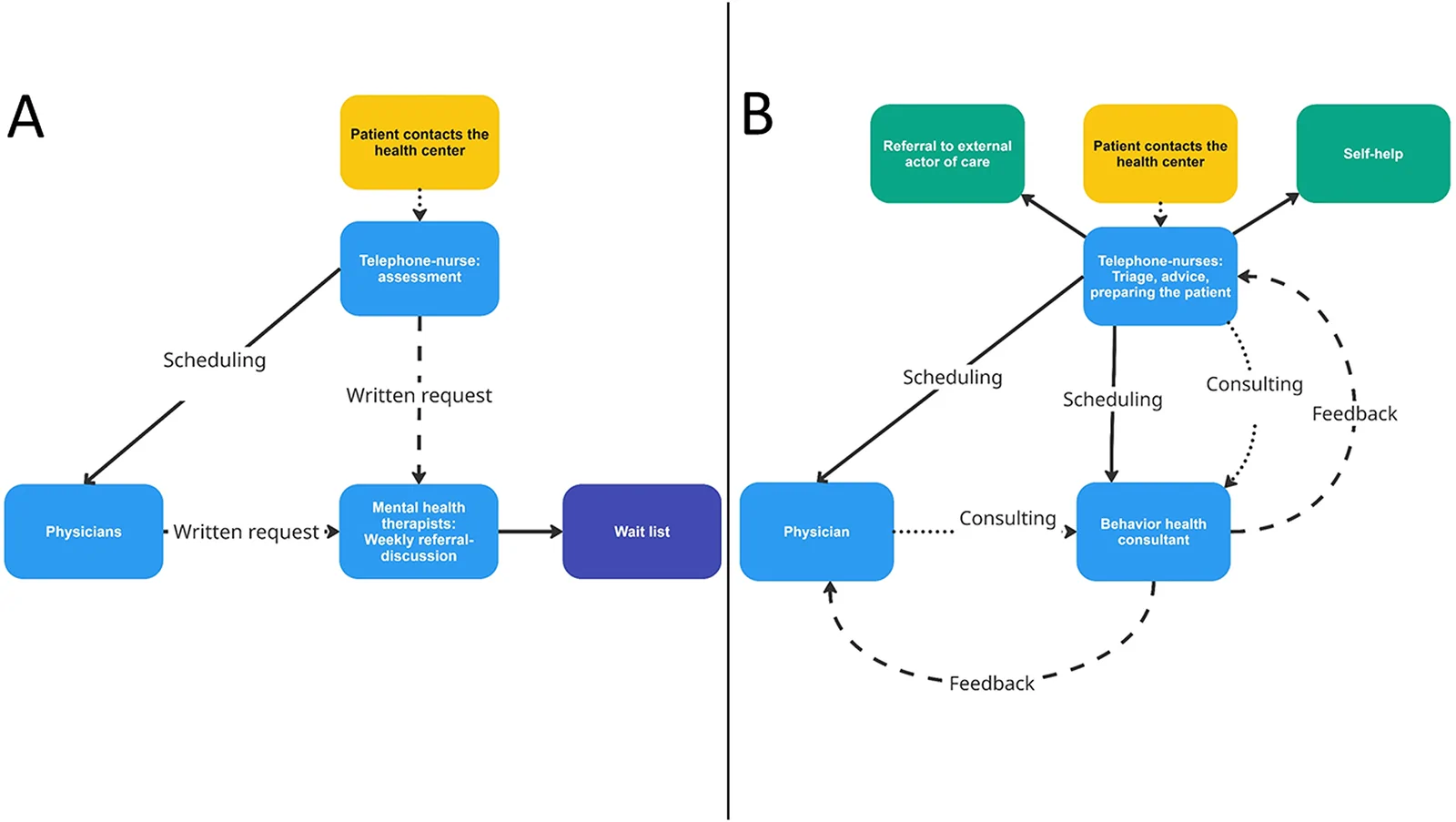
Clinical pathways before and after implementation of PCBH. Legends: **A** schematic picture of the clinical workflow before and after implementation of PCBH. Panel **A** shows the typical clinical workflow before implementation of PCBH, while panel **B** shows the typical clinical workflow after implementing PCBH

The integration of PCBH into routine practice in Region Östergötland was facilitated by a regional implementation team, consisting of psychologists with expertise in the PCBH model. In 2018, this regional implementation team planned implementation strategies, formed an action plan based on a quality implementation framework [23, 24], and created working materials and training based on previous literature on PCBH [22]. Simultaneously, information and planning meetings were held between the implementation team, stakeholders, decision-makers, managers, and other clinical representatives from all PCCs in the region. In 2019, the first four PCCs started the implementation process at each center. A more detailed description of the implementation process at each center is presented in Table 1, and the changes in clinical workflow are presented in Fig. 1.

**Table 1:** The implementation process at each health care center

Phase	Content	Time frame
Phase 1: initial assessment	For each PCC, interviews with key stakeholders were conducted to assess specific needs, available resources, readiness for change, organizational conditions, and implementation-related strengths and challenges. A local implementation team, comprising representatives from all professions, was established at each PCC to act as a link between the regional implementation team and the PCC employees. The local team served as key informants when setting the local objectives, activity scheduling, and evaluation timelines. Adaptations of both the intervention and organization were explored in collaboration between the regional and local implementation teams. Simultaneously, all clinical staff received customized PCBH training, ranging from 1 to 4 days: nurses attended 2 days, physicians 1 day, and BHCs 4 days. In addition, each profession was provided with tailored working materials specific to their role.	1–2 months
Phase 2: structure for implementation	In collaboration with the local implementation team, organizational changes were made to integrate PCBH into clinical routines. These included adjustments to the BHCs’ schedules, provision of materials to support PCBH workflows, and restructuring of workspaces to enhance teamwork among clinical staff. A plan outlining how and when PCBH implementation would begin was developed, and meetings for facilitation and supervision during the active implementation phase were scheduled. In addition, a structure for continuous feedback was established between the regional and local implementation teams, as well as between the regional team and PCC managers.	1–2 weeks
Phase 3: implementation	During the active implementation phase, the regional implementation team provided each center with facilitation support and supervision according to the pre-determined schedule, with additional support available on demand. Profession-specific methodologic guidance for working within the PCBH model was also offered. Meetings between the regional and local implementation teams were held every 4 weeks, and the regional team maintained regular contact with management. This approach allowed for early identification of each center’s needs, rapid adaptation of interventions, and real-time adjustments. Approximately 3 months into the active intervention phase, preliminary results and additional support needs were evaluated for each center. This evaluation was conducted collaboratively by the regional implementation team, the local implementation team, and individual groups of medical staff. A similar evaluation was performed 12 months after the active intervention phase, resulting in plans for continued maintenance, education, and follow-up tutorials tailored to each center. For most centers, this second evaluation indicated the need for an additional 6-month period of continuous support before transitioning to independent implementation of the PCBH model.	12–18 months
Phase 4: learn and improve	After the active implementation phase, implementation team meetings, voluntary group tutorials, and workshops were offered as maintenance activities. Profession-specific PCBH training was provided for new employees. On-demand support, along with workshops and lectures on PCBH-related topics, continued to be available. Based on feedback and outcomes from the pilot centers, the next implementation round was scheduled.	1 month ◊

The implementation was systematically executed in line with the first four steps of the Swedish Public Health Agency’s “Checklist for high-quality implementation” [23], which is based on the quality implementation framework [24]. Preparations at each health care center were made in phases 1 and 2, and the implementation started in phase 3. The implementation process for each center was scheduled to last approximately 12 months and was extended to 18 months in all centers. PCC, primary care center; PCBH, Primary Care Behavior Health; BHC, behavioral health consultant

### Contextual adaptations

The PCBH model was adapted to a Swedish context. Nurses play a key role in triage and the initial evaluation of patients in Sweden, therefore a major adaptation was the creation of profession-specific education as well as training specifically targeting nurses. In addition, stakeholders decided that the PCBH model would primarily target patients with behavioral and mental health concerns; interventions targeting solely somatic conditions were not included. Two general adaptations were also made regarding the use of focused BHC meetings and warm hand-offs. In Swedish primary care, there is a legislative requirement to provide evidence-based treatments for mild to moderate psychiatric disorders. To account for this, even though included centers implemented the brief, focused 15–30-minute appointments advocated in the original PCBH model, the BHCs retained the possibility to offer extended sessions of 45–60 min when clinically warranted to ensure adequate treatment. This flexible approach replaced the previous routine use of longer appointments. In addition, all BHCs adjusted their schedules to allow triage nurses to schedule appointments directly. While all staff were educated in warm hand-offs and brief interventions, and all centers added this option to their clinical workflow, the focus of the implementation was changes in clinical pathways and workflows, i.e., focusing on the “what” rather than the “how”. Figure 1 provides a schematic picture of clinical pathways before and after implementation of PCBH. Warm hand-offs were also adjusted based on the amount of time scheduled for BHCs at each PCC. These adaptations were considered minor and necessary to enable the implementation.

### Data collection

Various forms of data were collected. Reports from the regional implementation team were collected regarding the implementation process and local adjustments. Regarding accessibility to care, registry data from the electronic medical records (EMRs) and internal regional registers were used. For the BHCs, all appointments were assessed; for the physicians, all appointments for patients diagnosed with a mental, behavioral, or neurodevelopmental disorder (i.e., F-diagnosis, F00–F99) according to ICD-10 (International Statistical Classification of Diseases and Related Health Problems 10th Revision) were assessed. Appointments were of four types: individual in-person appointments, group or team appointments (i.e., either several patients meeting with one caregiver in e.g. group therapy, or one patient meeting several caregivers at one appointment), phone appointments and digital appointments. Registry data were also used to assess prescription of psychotropic drugs, defined as drugs with the following ATC (Anatomical Therapeutic Chemical) codes: N05A (antipsychotics), N05B (anxiolytics), N05C (hypnotics and sedatives) and, N06A (antidepressants).

Data were collected at baseline, set at 2 years before the start of implementation (period 1: 1 May 2017 to 30 April 2018), during the active intervention (period 2: 1 May 2019 to 30 April 2020), and the first year after implementation (period 3: 1 May 2020 to 30 April 2021). The baseline was set at 2 years before the start of implementation because information meetings regarding the PCBH model were being held in the region in 2018, and thus some of the pilot centers may have already started changing their way of working according to the principles of PCBH before the structured implementation was carried out.

### Participants

This study includes the first four PCCs that registered interest in working in accordance with the model and started their implementation in 2019. Details of each PCC are presented in Table 2. All patients 18 years or older receiving care according to the following inclusion criteria were included regarding registry data. Inclusion criteria: 18 years or older; within the periods of data collection, patients who had an in-person appointment with a BHC or had an in-person visit to a physician and diagnosed with mental, behavioral or neurodevelopmental health problem (defined by an F-diagnosis [F00–F99] according to ICD-10) or were prescribed psychotropic drugs (ATC codes: N05A, N05B, N05C, and N06A).

**Table 2:** Description of the primary health care centers included in the study

		Period 1	Period 2	Period 3
Unit A	Number of patients	14,676	14,768	15,033
	Number of full-time physicians	7.5	7.8	9.3
	Number of full-time BHC	3	3	2,6
Unit B	Number of patients	6733	6880	6993
	Number of full-time physicians	5.5	5.5	6.5
	Number of full-time BHC	0.7	1	1
Unit C	Number of patients	8886	9014	9129
	Number of full-time physicians	3	4,25	4,25
	Number of full-time BHC	0.6	1.0	1,0
Unit D	Number of patients	6189	6440	6477
	Number of full-time physicians	7.3	5.8	6.5
	Number of full-time BHC	0.5	0.5	0.75

Number of listed patients and staffing per primary care setting during baseline (period 1), during the active implementation period (period 2), and 1 year after the active implementation period started (period 3). All numbers are taken from the end of December in the time period in question. The number of patients was taken from internal registers; the number of full-time physicians and BHCs was estimated by the head of the unit. BHC, behavioral health consultant

**Fig. 2:**
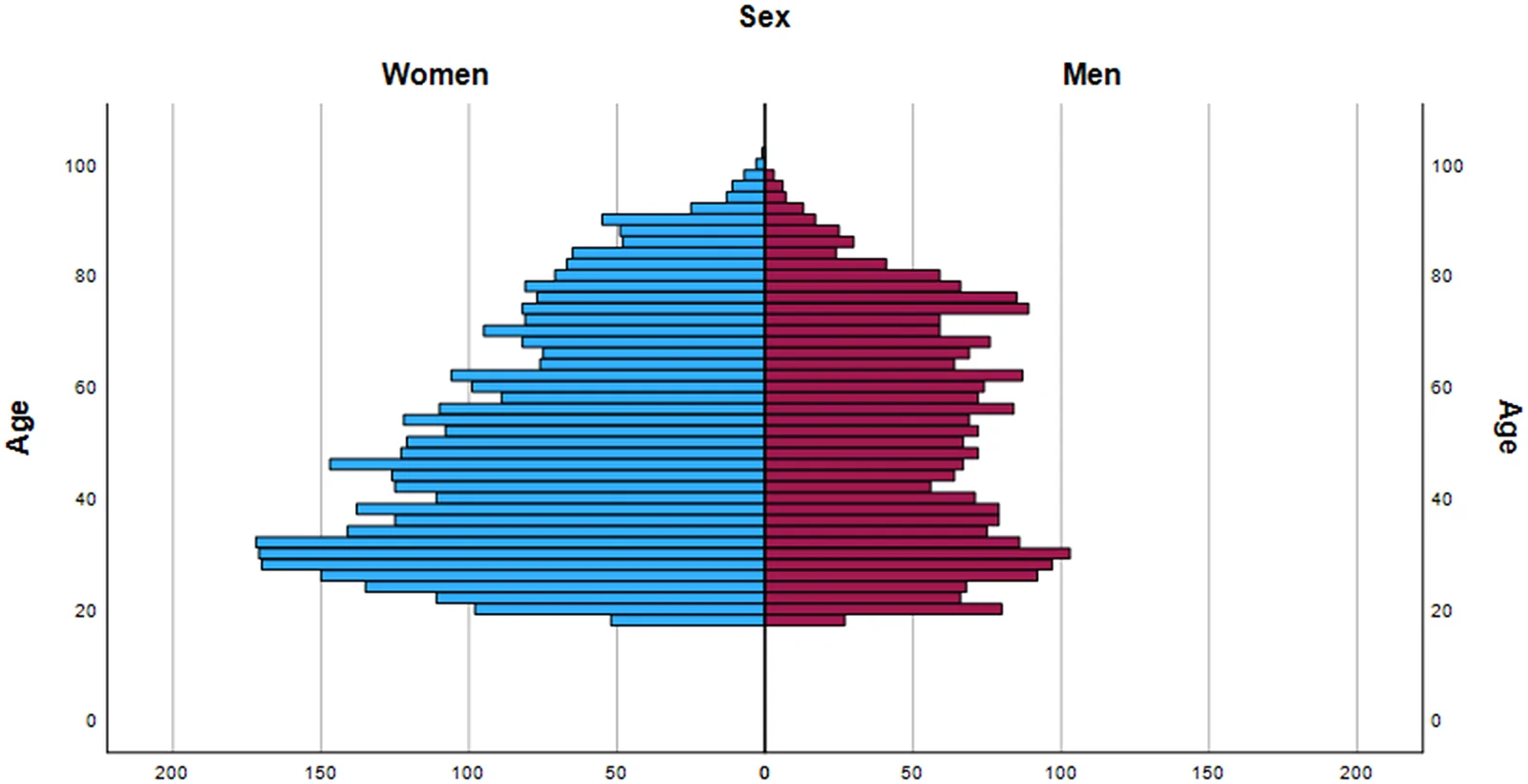
Gender and age distribution of participants

A total of 6413 individual patients were included in the study; 3914 (60%) women and 2599 (40%) men. The mean age was 49.8 years (standard deviation, 20.1 years, range 18–101 years; Fig. 2).

### Statistics

Data from EMRs were collected and directly transferred from the computerized system of Region Östergötland into Stata software (StataCorp, College Station, TX, USA). Data were then compiled into two separate sets: one containing diagnoses at visits and one containing prescriptions of selected psychotropic drugs. Statistical analyses were conducted using Stata v. 17.0; the significance level was set at *p* < 0.05. The waiting time for the first appointment was calculated from the initial contact with the PCC by an individual meeting the inclusion criteria to their first in-person appointment with a physician or BHC. The overall waiting time for appointments was calculated as the mean waiting time for all in-person visits during each period. Outliers, defined as waiting times exceeding 100 days (outliers period 1 *n* = 217 (total no. of observations: 4147); outliers period 2 *n* = 220 (total no. of observations: 5194); outliers period 3 *n* = 191 (total no. of observations: 6999)), were excluded from the analyses to reduce skewness. Waiting times below 0 days were interpreted as coding errors and also excluded from the analyses (period 1 *n* = 17; period 2 *n* = 9; period 3 *n* = 4). There were also some coding errors in the data from the EMRs regarding first visits and subsequent follow-up visits; for some patients, all appointments were coded as first visits, and for some, all appointments were coded as follow-up visits. Therefore, during data processing, the first visit each year for all included individuals was re-labeled as a new visit, and all subsequent visits that year were re-labeled as follow-up visits. This was justified due to clinical experience where the clinical average episode of care is < 1 year. Comparisons between different periods regarding waiting times (mean waiting times and waiting times for the first appointment) were performed using one-way ANOVA, with multiple comparisons adjusted using Bonferroni correction. Differences between periods in the number of appointments and individual patients were analyzed using a Z test (one-sample test of proportions), with adjustments for multiple comparisons using Bonferroni correction. When the number of observations was low (< 100) a binomial probability test was used instead, with adjustments for multiple comparisons by Bonferroni correction. When analyzing the care availability, only the individual in-person appointments were analyzed in-depth. This was done for two reasons; first, the in-person appointments consisted of the majority of all appointments, second, since many health care system pivoted to telehealth or digital visits during the pandemic (period 2 and 3), we wanted to exclude these types of appointments to avoid overinterpretation of the data.

## Results

### Appointments

#### All in-person appointments

Descriptive data regarding all appointments are provided in Table 3. When further analyzing the in-person appointments with a BHC at the pilot centers, these increased by 34% when comparing the baseline (period 1) with the active intervention phase (period 2) (*p* < 0.001). The year after the active intervention phase (period 3), the number of in-person appointments decreased compared with the intervention phase but was still increased by 21% from the baseline (*p* < 0.001). The total number of all in-person appointments with a physician for a mental, behavioral or neurodevelopmental health problem at the participating centers decreased by 4% (non-significant) when comparing period 2 with period 1, and then continued to decrease by 22% when comparing period 3 with period 1 (*p* < 0.001) (Table 4).

**Table 3:** Description of all appointments for mental health problems to BHC and physicians at the included centers

Patients and appointments	Period 1	Period 2	Period 3
**BHC**			
Total number of appointments	2184	3174	3852
In-person appointments:			
Individual patient appointments	1762	2359	2127
Group or team appointments	25	39	17
Remote appointments:			
Phone appointments	3	223	413
Digital appointments	394	553	1295
**Physicians**			
Total number of appointments	1543	1704	2243
In-person appointments:			
Individual patient appointments	1542	1476	1208
Group or team appointments	1	0	8
Remote appointments:			
Phone appointments	0	228	1019
Digital appointments	0	0	8

Period 1 refers to baseline, period 2 refers to the active implementation phase, and period 3 refers to the first year after the active implementation phase started. The group or team appointments refers to either several patients meeting with one caregiver in e.g. group therapy, or one patient meeting several care givers, a team, at one appointment), BHC, behavioral health consultant

**Table 4:** In-person appointments and individual patients receiving care for mental health problems

	Period 1	Period 2			Period 3				
	*n*	*n*	Period 2 versus period 1		*n*	Period 3 versus period 1		Period 3 versus period 2	
			% difference^a^	*p* value		% difference	*p* value	% difference	*p* value
**In-person appointments**									
With a BHC	1762	2359	+ 34	< **0.001**	2127	+ 21	**< 0.001**	−10	**< 0.001**
With a physician	1542	1476	−4	0.23	1208	−22	**< 0.001**	−18	**< 0.001**
**Individual patients**									
Seeing a BHC	702	995	+ 42	**< 0.001**	815	+ 16	**0.003**	−18	**< 0.001**
Seeing a physician	1050	966	−8	0.06	751	−28	**< 0.001**	−22	**< 0.001**
Only seeing a BHC	232	382	+ 65	**< 0.001**	312	+ 34	**< 0.001**	−18	**0.008**
Only seeing a physician	729	577	−20	**< 0.001**	448	−39	**< 0.001**	−22	**< 0.001**
Seeing both a BHC and a physician	321	389	+ 21	**0.01**	303	−6	0.47	−22	**0.001**

Period 1 refers to baseline, period 2 refers to the active implementation phase, and period 3 refers to the first year after the active implementation phase started. The % difference refers to the direction of the percentual difference between the time periods in question. Values in bold type are statistically significant

BHC, behavioral health consultant

^a^The + and − signs regarding % difference refer to the direction of the difference between the time periods in question

#### Individual patients

The number of individual patients who had an in-person appointment with a BHC showed a similar pattern as the appointments and increased by 42% when comparing period 1 with period 2 (*p* < 0.001). In period 3, the number of individual patients who had an appointment with a BHC decreased by 18% compared with period 2, i.e., a 16% increase compared to period 1 (*p* = 0.003). The number of individual patients who had an in-person appointment with a physician and were diagnosed with a mental, behavioral or neurodevelopmental health problem decreased by 8% when comparing period 2 with period 1 (non-significant), and then continued to decrease by 28% when comparing period 3 with period 1 (*p* < 0.001). The 22% reduction in the number of patients who saw a physician between periods 2 and 3 was also significant (*p* < 0.001). The number of individual patients who only saw a BHC increased in both period 2 and period 3 compared with baseline (an increase of 65% and 34%, respectively, *p* < 0.001 for both). The number of individual patients who only saw a physician decreased in both period 2 and period 3 compared with baseline (a decrease of 20% and 39%, respectively, *p* < 0.001 for both) (Table 4).

### Waiting times

#### All appointments

The mean waiting time for all in-person appointments with a BHC decreased by 24% when comparing period 1 with period 2 (*p* < 0.001). The reduction in mean waiting time remained stable during period 3 at 21% for all appointments (*p* < 0.001). The mean waiting time for all in-person appointments with a physician for a mental, behavioral or neurodevelopmental health problem increased by 20% when comparing period 1 with period 2 (*p* > 0.001), and this increase remained stable during period 3 at 22% compared with baseline (*p* < 0.001). There were no significant differences between periods 2 and 3 for BHCs or physicians (Table 5).

**Table 5:** Mean waiting time in days for in-person appointments with behavioral health consultants and physicians

	Period 1	Period 2			Period 3				
			Period 2 vs. period 1			Period 3 vs. period 1		Period 3 vs. period 2	
	Mean no. of days (SD) (no. of observations)	Mean no. of days (SD) (no. of observations)	% diff.	*P* value	Mean no. of days (SD) (no. of observations)	% diff.	*P* value	% diff.	*P* value
**Behavioral health consultants**									
All appointments	18.0 (14.7) (*n* = 1762)	13.7 (12.2) (2359)	-24%	**< 0.001**	14.2 (12.3) (*n* = 2127)	-21%	**< 0.001**	+ 4%	0.44
First appointment	15.8 (15.4) (*n* = 376)	9.7 (9.7) (*n* = 646)	-39%	**< 0.001**	11.0 (11.7) (*n* = 547)	-30%	**< 0.001**	+ 13%	0.16
**Physicians**									
All appointments F-diagnosis	11.0 (13.6) (*n* = 1542)	13.2 (18.0) (*n* = 1476)	+ 20%	**< 0.001**	13.4 (16.8) (*n* = 1208)	+ 22%	**< 0.001**	+ 2%	> 0.99
Physicians first appointment F-diagnosis*	10.0 (13.0) (*n* = 933)	11.7 (17.3) (*n* = 816)	+ 17%	0.051	11.4 (15.4) (*n* = 620)	+ 14%	0.98	-3%	0.18

Period 1 refers to baseline, period 2 refers to the active implementation phase, and period 3 refers to the first year after the active implementation phase started. Values in bold type are statistically significant

% Diff, Percentual difference. SD, standard deviation. Vs, versus. * mental, behavioral or neurodevelopmental health problems (F.00-F.99)

#### First appointment

The mean waiting time for the first in-person appointment with a BHC (the first visit after the person contacted the PCC with a mental or behavioral health problem) decreased by 39% when comparing the baseline with the active implementation phase, and the reduction remained relatively stable for the following year (a reduction of 30% compared with baseline). The differences between period 1 and periods 2 and 3 were significant (*p* < 0.001). There were no significant differences when comparing periods 2 and 3. There were no significant differences between the periods regarding waiting times for the first in-person visit with a physician for individuals diagnosed with a mental, behavioral or neurodevelopmental health problem (Table 5).

### Psychotropic drugs

#### Prescriptions

The total number of prescriptions of psychotropic drugs decreased by 18% between period 1 and period 2. Between period 2 and period 3, the total number of prescriptions increased by 5% (*p* = 0.004), but there was still a decrease of 14% compared with baseline (*p* < 0.001). Regarding the individual psychotropic drug classes, there were fewer prescriptions of all N05 drugs (antipsychotics, anxiolytics, hypnotics, and sedatives) during and after the active implementation phase of PCBH (*p* < 0.001 all comparisons). The number of prescriptions of N06 drugs (antidepressants) was similar between period 1 and period 2 but increased by 11% during period 3 (*p* = 0.01) (Table 6).

**Table 6:** Prescription of psychotropic medication

		Period 1	Period 2			Period 3				
		n	*n*	Period 2 versus period 1		n	Period 3 versus period 1		Period 3 versus period 2	
				% difference^a^	*P* value		% difference	*P* value	% difference	*P* value
Total^b^	Prescriptions	8025	6570	−18	**< 0.001**	6927	−14	**< 0.001**	+ 5	**0.004**
	Individuals	3388	3178	−6	**0.02**	3368	−1	> 0.99	+ 6	**0.04**
Antipsychotics (ATC: N05A)	Prescriptions	85	38	−55	**< 0.001**	35	−59	**< 0.001**	−8	> 0.99
	Individuals	68	36	−47	**0.004**	28	−59	**< 0.001**	−22	0.64
Anxiolytics (ATC: N05B)	Prescriptions	1956	1355	−31	**< 0.001**	1395	−29	**< 0.001**	+ 3	0.90
	Individuals	1018	773	−24	**< 0.001**	799	−22	**< 0.001**	+ 3	> 0.99
Hypnotics and sedatives (ATC: N05C)	Prescriptions	3122	2195	−30	**< 0.001**	2307	−26	**< 0.001**	+ 5	0.20
	Individuals	1339	1089	−19	**< 0.001**	1144	−15	**< 0.001**	+ 5	0.49
Antidepressants (ATC: N06A)	Prescriptions	2862	2982	+ 4	0.24	3190	+ 11	**< 0.001**	+ 7	**0.02**
	Individuals	2011	2057	+ 3	0.96	2247	+ 12	**< 0.001**	+ 9	**0.01**

Period 1 refers to baseline, period 2 refers to the active implementation phase, and period 3 refers to the first year after the active implementation phase started. Values in bold type are statistically significant

^a^The + and − signs regarding % difference refer to the direction of the difference between the time periods in question

^b^Total is a summary of all psychotropic prescriptions (N05A + N05B + N05C + N06A), further specified in the table

#### Individual patients

The number of individual patients who received a prescription for any psychotropic drug decreased by 6% during the first year of implementation compared with baseline (*p* = 0.02), but this number was the same as baseline by period 3. However, fewer individuals were prescribed any N05 drug (antipsychotics, anxiolytics, hypnotics, and sedatives) both during and after the active implementation phase of PCBH compared with baseline (*p* < 0.001 all comparisons), whereas the number of individuals who were prescribed any N06 drug was higher after the active implementation phase of PCBH (Table 6).

## Discussion

Despite limited evidence of its effectiveness, the PCBH model has generated considerable interest in primary care globally. Clinicians, decision-makers, and other stakeholders view the model as an appealing approach to organizing care, especially for patients with behavioral or mental health issues [25, 26]. The model is being implemented in several countries [20, 21, 27–29], emphasizing the need for further research on its effects. This pilot study presents the initial findings from a comprehensive research project evaluating a large-scale implementation of PCBH in a Swedish region [20]. The aim was to investigate the impact of integrating the model at four PCCs on waiting times and the use of mental health services provided by BHCs and physicians for adult patients. This study is an initial effort to assess the effectiveness of integrating the PCBH model into Swedish primary care. The results were promising, indicating increased accessibility to mental health services and a reduction in the prescription rates of psychotropic drugs.

This study demonstrated that, after the integration of the PCBH model, accessibility to mental health care provided by BHCs improved in the four PCCs. Specifically, this was shown as a decrease in the mean waiting time for in-person appointments with a BHC, and an increase in both the total number of appointments and the total number of individual patients receiving appointments with a BHC. The greatest improvements were observed in first visits, which was expected because the model aimed to shorten waiting times and reduce the queue for mental health care, which arises mainly before patients meet a BHC. Unfortunately the data did not allow for us to examine the percentage of individuals with same-day appointments. However, the result of shortened waiting times results are in agreement with one of the few previous studies regarding waiting times within the PCBH model, indicating that integrating PCBH into routine practice is associated with reduced waiting times and a higher number of patients receiving mental health care [16]. The increased number of patients appointed to a BHC could stem from several factors. One of the main reasons was changes in the schedules of BHCs. For example, nurses were able to book patients directly with the BHC, facilitating higher availability. Since the implementation of the PCBH model included education and the use of work sheets for triage nurses, it is also possible that more patients may have been found to have mental health concerns, even if that was not their reason to seek care. Another explanation could be that both nurses and physicians were able to detect more patients who would benefit from psychological rather than pharmacologic interventions. This hypothesis is supported by the decrease in overall prescription of psychotropic drugs observed throughout the study. This trend may suggest a shift towards the model’s foundation of biopsychosocial assessment and interventions not medicalizing behavioral concerns, possibly reducing the reliance on psychopharmacologic interventions [10]. Hypothetically, integrating the PCBH model into routine practice may have contributed to enhancing primary care professionals’ competence and awareness of mental health problems but further research is needed to examine this hypothesis. If this is the case, it would align with OECD recommendations for primary health care [8] and the original rationale for implementing PCBH in the southeast region of Sweden.

This study showed a reduction in the number of patients and appointments with physicians and longer waiting times to physicians for the target population. However, these results should not necessarily be interpreted as a lack of care during this period, and should be interpreted together with the observed increases in appointments with BHC’s. Fewer patients seeing a physician could likely be attributed to the new clinical pathway introduced by the model, whereby patients with these issues are first directed to see a BHC and begin with non-pharmacologic treatments, rather than the traditional method of initially seeing the physician. Thus, these results could probably be interpreted as adherence to the PCBH model.

Our investigation into the prescription of different classes of psychotropic drugs showed a decrease of prescription of psychotropic medication in total, and when divided in different drug classes, prescriptions for N05 drugs (antipsychotics, anxiolytics, hypnotics, and sedatives) decreased, while N06A (antidepressants) prescriptions increased. Lower rates of psychotropic prescriptions, may be considered a positive outcome if they reflect reduced use of potentially addictive drugs and more guideline-concordant prescribing, such as increased use of antidepressants when appropriate. Thus, the result of this study may reflect a preferred outcome, and may suggest that integrating the PCBH model into routine care helps change physicians’ prescribing habits, reducing reliance on the more addictive medications and encouraging the use of evidence-based treatments such as antidepressants. The training sessions for physicians highlighted the downsides of sedatives, particularly how they can interfere with important processes in psychological interventions, which may have contributed to this decrease. Another possible explanation is that part of the decrease of psychotropic prescription could be due to staff self-monitoring their prescribing as part of study participation as a PCC. Even though the majority of prescribing physicians were likely unaware or unaffected by the fact that their clinic was participating in a register-based study, we cannot rule out the possibility that better monitoring resulted in fewer people being prescribed a psychotropic drug, thus improving prescribing errors. At the same time, the increase in antidepressant prescriptions could be due to improved patient triaging. With better access to BHCs, physicians are likely to see patients with more severe symptoms or those explicitly requesting medication as a first-line treatment. Unfortunatelly, we were not able to pair the prescription data with individuals receiving care from a BHC, but the decreased prescription of potentially addictive psychotropic drugs together with an increase in number of patients with an appointment to a BHC, probably suggests a more systematic approach to matching patients with the right type of care, in line with Swedish guidelines that prioritize psychological therapies but recognize the importance of antidepressants when needed. Further in-depth research is needed to further explore the subject.

Although most of the differences in accessibility to care and the prescription of psychotropic drugs between the baseline and the active implementation period showed sustained improvement even after implementation, the results indicate a decline in positive outcomes over time. This decline may be attributed to several factors. It is possible that the population requiring mental health interventions in the initial period was larger than in the second period when the patient flow was more normal. Alternatively, the reduced number of implementation activities during this period influenced both the identification of patients in need of a BHC and recognition of the importance of avoiding medications for mild behavioral health symptoms. Or it could be that triaging nurses and other staff became more skilled at early detection of patients, recommending targeted self-care interventions, preventing a possible worsening of symptoms, and reducing the need for BHC appointments. Another possible explanation is that the restrictions after the start of the COVID-19 pandemic had an impact. The pandemic occurred during data collection (i.e., starting in December 2019) with nationwide restrictions from March 2020. The restrictions partially overlap with the end of period 2 and continue to overlap during parts of period 3, 1 year after the model had been integrated into regular practice. Remote appointments were advocated during restrictions, and to avoid the risk of over-interpretating the results, we chose to exclude all remote appointments for the in-depth analyses in this study and chose to only present them as descriptive data. The pandemic may also have had an impact on implementation, services, and patients’ willingness to seek healthcare. However, in-person appointments to a BHC still seemed to increase regardless of COVID-19, which rather strengthens the results. This also contrasts with the trend for other primary healthcare visits during the pandemic [30, 31], therefore the pandemic may have caused a type II error effect on the results, i.e., partially attenuating the true impact of implementing PCBH on care accessibility. Further research with more centers and longer follow-up times is needed to analyze this further. The possible impact of the pandemic, the impact of implementation activities, or staff knowledge and experiences were not analyzed in this study, yet it is possible that some or all of these factors affected the results. Therefore, the evidence from this study should be interpreted cautiously.

In the original PCBH model, a BHC engages with any sort of biopsychosocial concerns, ranging from medically unexplained symptoms to chronic diseases (e.g., diabetes) and mental health problems. When introducing the model in Region Östergötland, decision-makers and PCC managers decided to narrow the scope of the model to patients with mental health concerns only. This adjustment was intended to reduce the complexity of altered clinical pathways and benefit the acceptance of the PCBH model among the BHCs. However, a patient could still see a BHC regardless of the original reason for seeking care; i.e., if a patient with diabetes also had behavioral or mental health concerns, they could see a BHC. Regarding the implementation process, all PCCs made exceptions to the initial protocol set up by the regional implementation team concerning appointment duration, as previously described in the contextual adaptations. The PCBH model emphasizes that behavioral interventions are typically guided by the judgment of individual clinicians [16], therefore this is not considered a fidelity issue. Overall, relatively few contextual adaptations had to be made to adapt PCBH to Swedish primary care, and the feasibility and acceptability of the model were therefore suggested to be high. In addition, the model served as a lens through which all staff, not just BHCs, could approach patients with behavioral problems using a biopsychosocial framework.

### Strengths and limitations

The study has several limitations that must be considered when interpreting the findings. First, the four PCCs served as their own historical controls, and there were no control groups established during the same periods. In the larger trial we also aim to include comparison groups to study the effectiveness of PCBH compared to routine care instead of just before-after measurements. Second, the first four centers that integrated the PCBH model into routine practice had expressed interest in implementing the model. This may have introduced selection bias, because these PCCs might not be representative of primary care as a whole, thus limiting the generalizability of the findings. To control for generalizability, the larger study has been designed as a stepped-wedge cluster trial with a control group of matched PCCs [20]. Third, the COVID-19 pandemic occurred during the implementation period, which likely influenced the implementation. Forth, since the implementation of PCBH in Swedish primary care mainly targeted patients with behavioral and mental health concerns, and BHCs retained the possibility of offering care appointments of 45–60 min according to patients’ needs, this may reduce generalizability to standard PCBH implementations. However, the care format of brief, focused visits advocated in the original PCBH model was implemented at all centers, extended sessions were used only when clinically necessary and not as standard, and the model was applied broadly within the context of routine primary care. We therefore do not expect this to have substantially influenced the overall applicability of the findings. Other limitations include the inability to link individual patients to specific prescriptions, which means that we cannot entirely rule out the possibility that reduced prescription rates are influenced by patient dropout. Neither can we rule out that part of the results regarding prescription pattern may be due to self-monitoring and improving prescribing errors. Nevertheless, the study’s population-based design, its setting in routine primary care, and the use of retrospectively collected data from regional healthcare registers where the majority of prescribing physicians at the time for prescription were likely not aware of the fact that their clinic was participating in a register-based study, likely mitigate these potential sources of bias.

The study also has important strengths. First, there are very few published clinical trials [16, 32, 33] and only one other study protocol of a pragmatic clinical trial [21] regarding PCBH. The larger study of which this pilot trial is part is one of the few multicenter clinical trials that aim to investigate the effects of integrating PCBH into routine primary care. Second, the study design emphasized external validity, i.e., studying real-world effects instead of effects under ideal, controlled conditions, specifically in this pilot trial the association of PCBH on access to behavioral health care in routine primary care. Third, the data concerning accessibility to care as well as prescription of psychotropic drugs were collected prospectively from registry data. Thus, these results are robust because data from Swedish medical files have previously been described as highly reliable. Data were also collected at multiple time points, both before facilitation started and after the integration of the work model in the PCCs, providing a solid comparison of trends over time. The inclusion of multiple cases further enhances the generalizability of the findings across various settings, offering a comprehensive view of the potential of the PCBH model. Fifth, the project was developed and carried out in close cooperation with primary care staff members and decision-makers, contributing to external validity and the applied clinical nature of the project.

## Conclusions

After integration of the PCBH model into routine practice at four PCCs in southeast Sweden, access to mental health care improved, and the prescription of psychotropic drugs decreased. We recommend that further research should include more centers with diverse prerequisites and long-time follow-up to ensure generalizability of the findings, as well as adequate comparison groups. We also recommend systematically incorporating patient-reported measures to assess the impact on patient outcomes and satisfaction, as well as staff experiences of implementing and working according to the model. With proper measurement of patient functioning, an in-depth analysis of cost-effectiveness could also be included, adding to the knowledge of the model. In addition, research should consider the experiences of healthcare professionals because they are key stakeholders in the implementation and maintenance of the model [34].

## Data Availability

The authors confirm that all data supporting the findings of this study are available within the paper.

## Abbreviations

ATC: Anatomical Therapeutic Chemical
BHC: Behavioral Health Consultant
EMR: Electronic Medical Records
ICD-10: International Statistical Classification of Diseases and Related Health Problems 10th Revision
OECD: The Organization for Economic Co-operation and Development
PCBH: Primary Care Behavioral Health; PCC: primary care centers
